# Cell-Based Immunotherapy With Mesenchymal Stem Cells Cures Bisphosphonate-Related Osteonecrosis of the Jaw–like Disease in Mice

**DOI:** 10.1002/jbmr.37

**Published:** 2010-01-29

**Authors:** Takashi Kikuiri, Insoo Kim, Takyoshi Yamaza, Kentaro Akiyama, Qunzhou Zhang, Yunsheng Li, Chider Chen, WanJun Chen, Songlin Wang, Anh D Le, Songtao Shi

**Affiliations:** 1 Center for Craniofacial Molecular Biology, University of Southern California School of DentistryLos Angeles, CA, USA; 2 Salivary Gland Disease Center and the Molecular Laboratory for Gene Therapy and Tooth Regeneration, Capital Medical University School of StomatologyBeijing, people's Republic of China; 3 National Institute of Dental and Craniofacial Research, National Institutes of HealthBethesda, MD, USA

**Keywords:** mesenchymal stem cell, osteonecrosis, bisphosphonate, treg, TH17, interleukin 17

## Abstract

Patients on high-dose bisphosphonate and immunosuppressive therapy have an increased risk of bisphosphonate-related osteonecrosis of the jaw (BRONJ); despite the disease severity, its pathophysiology remains unknown, and appropriate therapy is not established. Here we have developed a mouse model of BRONJ-like disease that recapitulates major clinical and radiographic manifestations of the human disease, including characteristic features of an open alveolar socket, exposed necrotic bone or sequestra, increased inflammatory infiltrates, osseous sclerosis, and radiopaque alveolar bone. We show that administration of zoledronate, a potent aminobisphosphonate, and dexamethasone, an immunosuppressant drug, causes BRONJ-like disease in mice in part by suppressing the adaptive regulatory T cells, Tregs, and activating the inflammatory T-helper-producing interleukin 17 cells, Th17. Most interestingly, we demonstrate that systemic infusion with mesenchymal stem cells (MSCs) prevents and cures BRONJ-like disease possibly via induction of peripheral tolerance, shown as an inhibition of Th17 and increase in Treg cells. The suppressed Tregs/Th17 ratio in zoledronate- and dexamethasone-treated mice is restored in mice undergoing salvage therapy with Tregs. These findings provide evidence of an immunity-based mechanism of BRONJ-like disease and support the rationale for in vivo immunomodulatory therapy using Tregs or MSCs to treat BRONJ. © 2010 American Society for Bone and Mineral Research.

## Introduction

Over the past 30 years, bisphosphonates (BPs), a major class of anti–bone resorptive drug, have been used routinely to manage skeletal complications associated with osteoclast-mediated bone loss in osteoporosis, primary osteolytic pathology of bone such as Paget disease and multiple myeloma,(1,2) and complications of metastatic diseases.(3,4) Administration of BPs in these patients effectively restores bone mineral density (BMD) and bone strength, reduces the incidence of bone fracture, and dramatically improves the quality of life. However, the recent recognition that BP use is associated with pathologic conditions, including BP-related osteonecrosis of the jaw (BRONJ), has sharpened the level of scrutiny of the current widespread use of BP therapy.(5–7)

BRONJ is a newly described condition defined as exposed necrotic bone in the maxillofacial region that fails to heal after 8 weeks in patients with no history of craniofacial radiation.(8) The majority of BRONJ cases (94%) have been reported in patients receiving high doses of intravenous (iv) aminobisphosphonates (primarily zoledronic acid and pamidronate) for oncologic conditions.(9) Prevalence in patients with multiple myeloma ranged from 7% to 10%, whereas up to 4% of patients with breast cancer developed BRONJ.(8,9) The estimated incidence of BRONJ in patients receiving intravenous BPs for malignant disease ranges from 0.8% to 12%,(10,11) an incidence 900-fold higher than that of general osteoporosis patients.(12) The doses for oncologic indications of BP are 10 to 12 times higher than those used in osteoporosis(2,13) and usually in combination with steroids.(14,15) Risk factors for BRONJ include invasive dental procedures, infections, mechanical trauma to the jawbone, and length of exposure to BPs, as well as concomitant use of immunosuppressive and chemotherapy drugs.(8,9) Since most of the cancer patients were on multiple immunosuppressant drugs, including dexamethasone (Dex) and chemotherapeutic agents, and therefore experienced some degree of impaired immunity, it is likely that immunosuppression contributes to an increased susceptibility to BRONJ.

Patients with BRONJ present various jaw symptoms, including pain, swelling, infection, loose teeth, and exposed bones in some severe cases.(1–4) Histologically, BRONJ manifests in diverse tissue changes, including necrotic bone honeycombed with residual vital bone, inflammatory cellular elements, and fibrous tissues.(8,9,12) Most attempts to control this disorder have not been successful, and standard osseous sequestrectomy usually results in further enlarging the bony defects.(16,17) Therefore, conservative nonsurgical approaches have been recommended in managing BRONJ patients that only slow down the deterioration but do not cure the disease.(18–20) To develop an effective approach to prevent and treat BRONJ becomes an urgent issue for patients using BPs.

Here we describe a mouse model of BRONJ that recapitulates major clinical and radiographic manifestations of the human disease,(8,9,12) including its characteristic features of delayed healing displayed orally as an open alveolar socket without mucosal coverage, exposed necrotic bone or sequestra, increased inflammatory infiltrates, osseous sclerosis, and radiopaque alveolar bone in the jaw. In this study we have demonstrated that zoledronate (Zol) and Dex can cause BRONJ-like lesion in mice following tooth extraction. We show that administration of Zol and Dex causes BRONJ in part by suppressing the adaptive regulatory T cells, Tregs, and increasing the inflammatory T-helper subset cells, Th17, producing interleukin 17 (IL-17) in peripheral blood. The alteration in the balance between Tregs/Th17 was observed in Zol/Dex-treated mice and was restored in mice that underwent salvage therapy to replenish Tregs or pan-T cells. More importantly, cell-based therapy using systemic mesenchymal stem cell (MSC) infusion can prevent or cure BRONJ via reestablishment of the immune balance between Treg and Th17 cells, as seen in the Treg-treated group. Understanding how the immunologic balance between Treg/Th17 cells alters the inflammatory response of alveolar socket healing is critical in further elucidating the pathogenesis of BP-induced osteonecrosis of the jawbones and will open a new avenue for research into the mechanism and, ultimately, preventive and therapeutic interventions of human BRONJ diseases.

## Materials and Methods

### Animals

C57BL/6J mice (female, 8 to 10 weeks old; Jackson Laboratory, Bar Harbor, ME, USA) and beige nude/nude Xid (III) mice (female, 8 to 10 weeks old; Harlan, Indianapolis, IN, USA) were used in this study, as described in the Supplementary Information. All animal experiments were performed under an institutionally approved protocol for the use of animal research at the University of Southern California (USC) (USC 10874 and 10941).

### Antibodies

All antibodies used in this study are described in the Supplementary Information.

### Generation of BRONJ-like mouse model

Mice received intravenous zoledronate (Zometa, 125 µg/kg; Novartis Oncology, East Hanover, NJ, USA) and/or dexamethasone (5 mg/kg; Sigma, St. Louis, MO, USA) twice a week via the tail vein. One week after iv injection, maxillary first molars were extracted under deep anesthesia by ip injection of ketamine (Ketaject, 35 mg/kg; Phoenix, St. Joseph, MO, USA) and xylazine (Xylaject, 5 mg/kg; Phoenix). Then 2 and 7 weeks after tooth extraction, the intact maxillas were harvested en bloc. A total of 6 and 16 doses of zoledronate and dexamethasone were administered for the 2- and 7-week follow-up groups, respectively. Untreated mice with tooth extraction or mice without tooth extraction were used as controls. In parallel, peripheral blood was collected for T cell and cytokine analyses. Details regarding sample preparation are provided in the Supplementary Information.

### Adaptive transfer of T-lymphocytes in immunocompromised mice

Purification of pan-T cells, CD4^+^CD25^+^ regulatory T-lymphocytes (Tregs), and Tregs-depleted pan-T cells is described in the Supplementary Information. T-lymphocyte transfusion was performed as described previously.(21) See details in Supplementary Information.

### Treatment with neutralizing antibody

Anti-CD25 monoclonal antibody (PC61, 1 mg/mL) was injected ip once in C57BL/6J mice 2 days before tooth extraction. In parallel, mice received subclass-matched control antibody (1 mg per mouse), which served as control.

### Allogeneic mouse mesenchymal stem cell (MSC) transplantation

MSCs were isolated from bone marrow (BMMSCs) of femurs and tibias of C57BL/6J mice as reported previously(21) (see Supplementary Information). Under general anesthesia, BMMSCs (1 × 10^6^ cells/100 µL of saline per mouse) were infused iv in mice via tail vein 2 days after tooth extraction, as described previously.(22)

### Histomorphometry

Histomorphometric analysis was quantified as described previously.(23) Detailed methods are described in the Supplementary Information.

### Flow cytometric analysis

Flow cytometric staining and analysis were performed as reported previously(22) (see Supplementary Information).

### Statistical analysis

Statistical analyses were performed using Student's *t* test (Systat Software, Inc., Point Richmond, CA, USA). *p* values less than .05 were considered significant.

## Results

### Combination of bisphosphonate and dexamethasone treatment enhanced BRONJ-like disease in mice

**Fig. 1:**
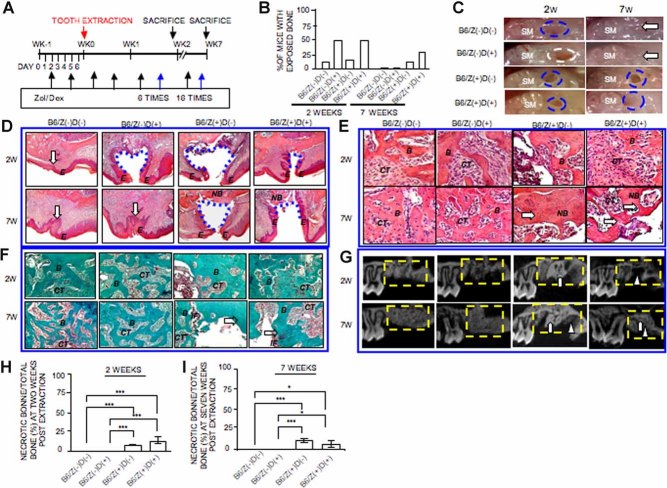
Development of BRONJ-like lesions in C57BL/6J mice. (*A*) Experimental protocol of inducing a BRONJ-like murine model. C57BL/6J (B6) mice were intravenously injected with Zol (125 µg/kg) and Dex (5 mg/kg) twice weekly for 1 week before surgical extraction of the left maxillary first molar and continuous injection of Zol and Dex twice weekly for 2 or 7 weeks. (*B*) Incidence of BRONJ-like lesion, manifested as an unhealed open socket with an area of exposed bone and no mucosal coverage, at the extraction site in B6 mice treated with Zol (Z) and Dex (D) (B6/Z^+^D^+^) for 3 and 8 weeks, whereby mice with no treatment (B6/Z^−^D^−^), Dex only (B6/Z^−^D^+^), and Zol only (B6/Z^+^D^−^) served as controls. (*C*) Representative gross clinical appearance of gingival mucosa at the extraction sites at 2 and 7 weeks after tooth extraction. Blue circles represent images of the apparent mucosal disruption with exposed bone at the extracted site adjacent to the second molar (SM), whereas open arrows point to the healed gingival mucosa. (*D*) Histologic images of extraction sockets showing open sockets without epithelial lining (*blue dot line*) and healed gingival mucosa with complete epithelial coverage (*open arrow*). NB = necrotic bone. (*E*, *F*) H&E (*E*) and trichrome (*F*) staining of extraction socket areas displaying newly formed bone (B), connective tissues (CT), necrotic bone (NB), and inflammatory cell infiltrates (IF); open arrows point to the necrotic bones. (*G*) µCT analysis showing necrotic bone (*open arrow*) and reduced bone formation (*triangle*) in B6/Z^+^D^+^ mice. (*H*, *I*) Quantification of necrotic bone areas in B6/Z^+^D^+^ and B6/Z^−^D^−^ groups at 2 and 7 weeks after tooth extraction. *p < .05; ***p < .005. The results are representative of three independent experiments and are shown as mean ± SD.

**Fig. 2:**
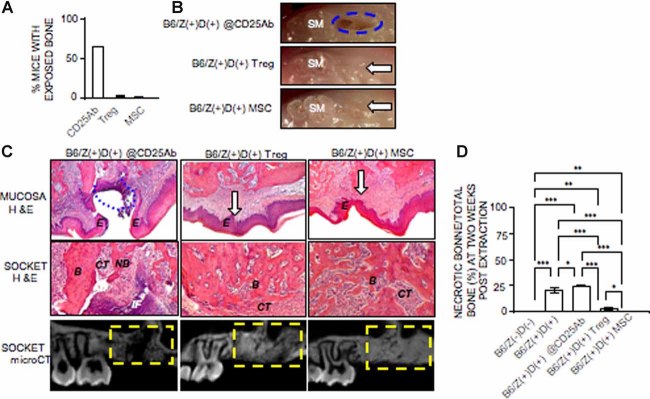
Treg and MSC treatment prevents development of BRONJ-like lesions and enhances new bone formation in the C57BL/6J mouse model. (*A*) Incidence of BRONJ-like lesions, manifested as an unhealed open socket with area of exposed bone and no mucosal coverage, at the extraction site in Zol/Dex-treated C57BL/6J mice receiving ip injections of anti-CD25 antibody (CD25Ab) (B6/Z^+^D^+^@CD25Ab) or systemic infusion of Tregs (B6/Z^+^D^+^Treg) or MSCs (B6/Z^+^D^+^MSC) at 7 weeks after tooth extraction. CD25Ab was administered 2 days before tooth extraction. Treg and MSC infusions were initiated 2 days after tooth extraction. (*B*) Representative gross clinical appearance of gingival mucosa at the extraction sites at 7 weeks after tooth extraction. Blue circles represent images of the apparent mucosal disruption, with exposed bone at the extracted site adjacent to the second molar (SM), whereas open arrows point to the healed gingival mucosa. (*C*) Histologic analysis showing an open socket without epithelial lining (*blue dot line*), healed gingival mucosa with complete epithelial coverage (*open arrow*) at the extraction sites (*upper panel*), and newly formed bone (B), connective tissues (CT), necrotic bone (NB), and inflammatory cell infiltrates (IF) (*middle panels*); open arrows point to the necrotic bones; µCT analysis (*lower panel*) showing necrotic bone and reduced bone formation in Zol/Dex-treated C57BL/6J mice receiving ip injections of CD25 antibody (B6/Z^+^D^+^@CD25Ab) but not in mice treated with systemic infusion of Tregs (B6/Z^+^D^+^Treg) or MSCs (B6/Z^+^D^+^MSC). (*D*) Quantification of necrotic bone areas in Zol/Dex-treated C57BL/6J mice receiving ip injection of CD25Ab or systemic infusion of Tregs and MSCs at 2 weeks after tooth extraction. *p < .05; **p < .01; ***p < .005. The results are representative of three independent experiments.

**Fig. 3:**
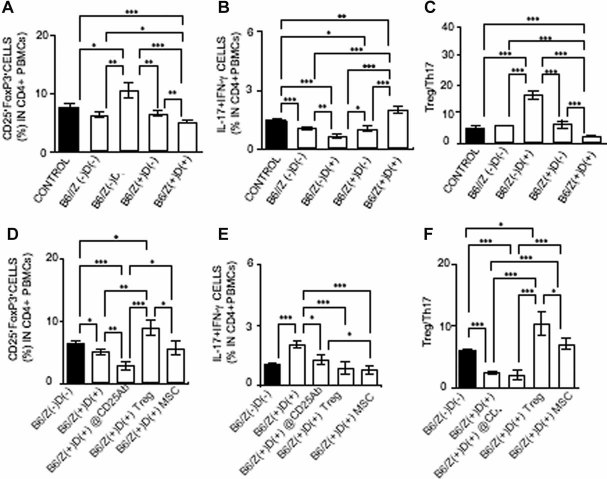
Effects of various treatments on T cell profile of CD4^+^CD25^+^Foxp3^+^ Tregs and CD4^+^IL17^+^IFNγ^−^ Th17 cells in BRONJ-like C57BL/6J mice. (*A*, *D*) Profile of CD4^+^CD25^+^Foxp3^+^ Tregs in peripheral blood of C57BL/6J mice treated with Zol (Z) and Dex (D) (B6/Z^+^D^+^) (*A*), Zol/Dex and ip injection of CD25Ab (B6/Z^+^D^+^@CD25Ab), Zol/Dex and systemic infusion of Tregs (B6/Z^+^D^+^Treg), or MSCs (B6/Z^+^D^+^MSC) (*D*) at 2 weeks after tooth extraction; mice with or without tooth extraction (control) and no drug treatment (B6/Z^−^D^−^), Dex only (B6/Z^−^D^+^), and Zol only (B6/Z^+^D^−^) served as controls. *p < .05; **p < .01; ***p < .001. (*B*, *E*) Profile of CD4^+^IL17^+^IFNγ^−^ cells in peripheral blood of C57BL/6J mice treated with Zol (Z) and Dex (D) (B6/Z^+^D^+^) (*B*), Zol/Dex and ip injection of CD25Ab (B6/Z^+^D^+^@CD25Ab), Zol/Dex and systemic infusion of Tregs (B6/Z^+^D^+^Treg) or MSCs (B6/Z^+^D^+^MSC) (*E*) at 2 weeks after tooth extraction. Mice with no tooth extraction (control) or with tooth extraction but with no treatment (B6/Z^−^D^−^) served as controls. *p < .05; **p < .01; ***p < .005. (*C*, *F*) The ratio of CD4^+^CD25^+^Foxp3^+^ cells (Tregs) and CD4^+^IL17^+^IFNγ^−^ cells (Th17 cells) in peripheral blood of C57BL/6J mice after receiving different treatment regimens. *p < .05; ***p < .005. The results are representative of three independent experiments.

**Fig. 4:**
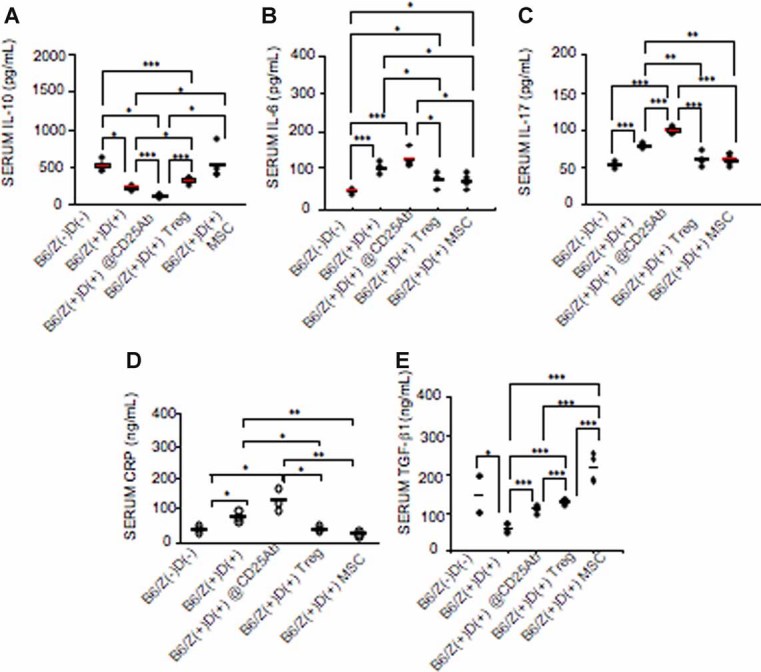
Effects of various treatments on serum inflammatory cytokine level in BRONJ-like C57BL/6J mice. C57BL/6J mice were treated with Zol (Z) and Dex (D) (B6/Z^+^D^+^), Zol/Dex and ip injection of CD25Ab (B6/Z^+^D^+^@CD25Ab), Zol/Dex and systemic infusion of Tregs (B6/Z^+^D^+^Treg), or MSCs (B6/Z^+^D^+^MSC) at 2 weeks after tooth extraction. Peripheral blood samples were collected, and serum levels of IL-10 (*A*), IL-6 (*B*), IL-17 (*C*), CRP (*D*), and total TGF-β1 (*E*) were determined by ELISA. Mice with no tooth extraction or with tooth extraction but with no treatment (B6/Z^−^D^−^) served as controls. *p < .05; **p < .01; ***p < .005. The results are representative of three independent experiments.

BRONJ has been correlated with high doses of nitrogen-containing BPs,(4) with an incidence of up to 18% in cancer patients treated with iv zoledronate or pamidronate for bone metastases in conjunction with immunosuppressive chemotherapy.(7) Based on these striking epidemiologic findings, we seek to adapt an equivalent treatment protocol of high-dose iv nitrogen-containing BP, zoledronate (Zol), and an immunosuppressive drug, dexamethasone (Dex),(18,19) in the development of a BRONJ-like disease model. Wild-type C57BL/6J mice were injected iv with Zol at 125 µg/kg of body weight and Dex at 5 mg/kg of body weight 1 week before extraction of the first maxillary molar, and the drug treatment continued twice a week until euthanazia time points (Fig. 1*A*). Control mice were treated with Dex alone or saline. Thereafter, the maxillary bones were evaluated for clinical signs of human BRONJ lesions, following the guidelines established by the American Association of Oral and Maxillofacial Surgeons (AAOMS) and the American Society for Bone and Mineral Research (ASBMR).^(5,17,24)^ Clinical examination at 2 weeks after surgical extraction (3 weeks after Zol/Dex treatment) revealed incomplete mucosal healing and presence of open sockets with exposed bone in 50% of mice treated with Dex only or the combination of Zol and Dex and 17% of mice treated with Zol only versus 11% of untreated control mice (Fig. 1*B, C*). The finding of open sockets with areas of exposed bone in these treated mice was further confirmed with histologic study revealing a lack of epithelial lining at the alveolar socket (Fig. 1*D*). In the untreated control mice, despite the apparent depressed mucosal surface above the alveolar socket, we observed a complete epithelial coverage in 89% of the mice, as shown by histologic study (Fig. 1*D*), suggesting a normal course of extraction socket healing within 2 weeks of extraction. At 7 weeks after surgical extraction, 100% of Dex-treated or untreated control mice healed with complete epithelial coverage, whereas 10% of Zol-treated and 30% to 33% of Zol- and Dex-treated mice failed to heal, resulting in exposed necrotic bone and bone sequestra at the extraction sockets (Fig. 1*B–D*). Histologic analysis showed the presence of increased inflammatory infiltrates and necrotic bone areas with empty lacunae, fibrosis, and lack of epithelial lining overlying areas of dead bone (Fig. 1*D, E*). We confirmed that mice treated with Zol only and the combination of Zol and Dex developed BRONJ-like lesions based on the following criteria: (1) presence of an open extraction socket with areas of exposed bone and without mucosal coverage that fails to heal within 2 weeks, the normal course of healing in nontreated control mice (Fig. 1*B–D*), (2) the presence of empty lacunae and necrotic bone or/and sequestra (Fig. 1*D–F*), (3) the presence of moderate infiltrate of inflammatory cells (Fig. 1*D–F*), and (4) the presence of radiopaque areas of the necrotized bone at the extracted socket (Fig. 1*G*). We found that bone necrosis occurred exclusively within the extracted alveolar socket site because the distal bone was unaffected (Figs. 1*D–F*). Moreover, most of the necrotic bone was found adjacent to the area of intense local inflammatory infiltrates, suggesting an association between inflammation and tissue degeneration/necrosis in BRONJ-like disease (Fig. 1*D–F*). As expected, the extraction sockets in control animals without Zol/Dex treatment underwent a regular course of healing with rapid epithelialization within 2 to 3 weeks as well as bone regeneration (Figs. 1*B–G*). Radiographic evaluation by micro–computed tomography (µCT) showed the presence of a poorly defined alveolar ridge owing to impaired bone healing and a mottled trabecular pattern with mixed radiopaque/radiodense areas of bone necrosis in the intrabony alveolar sockets of mice treated with Zol only and the combination of Zol and Dex (Fig. 1*G*). To quantify bone necrosis, we digitally scanned hematoxylin and eosine (H&E)–stained sections of the extracted alveolar sockets using a ScanScope slide scanner (Aperio Technologies, Vista, CA, USA) and analyzed areas of dead bone using the ImageScope software from Aperio. The total number of lacunae within a defined region was counted manually, and the percentage of empty lacunae was calculated for each section. Bone necrosis was defined as three or more empty lacunae per 1000 µm^2^. An average percentage of necrotic bone area over the total area of bone was calculated (Fig. 1*H, I*). Clearly, Zol alone and the combination of Zol and Dex treatment could induce bone necrosis at 2 and 7 weeks after tooth extraction (Fig. 1*H, I*). The incidence of BRONJ was consistently higher in mice treated with the combination of Zol and Dex compared with Zol alone, suggesting that immunosuppressive therapy may render mice more susceptible to the BRONJ lesion. We next examined the effect of Zol/Dex treatment on osteoclast levels via TRACP staining (Supplemental Fig. 1*A*) and observed a marked suppression of osteoclast activities in all Zol/Dex-treated mice, confirming its well-established antiresorptive property.

Taken together, the current BRONJ-like murine model allows the first direct evidence that the cumulative high dose of Zol is the etiologic cause of BRONJ-like disease in mice undergoing dental extraction during the course of BP treatment. In agreement with human epidemiologic studies, we also observed a relatively low incidence of BRONJ in the Zol-only treated group that escalated with the addition of an immunosuppressive therapy, a parallel analogy to cancer patients on chemotherapy or steroid therapy.(15,16)

### Cell-based immunotherapy restored both mucosal and bone healing at the extracted alveolar socket

A systematic review of reported cases of BRONJ from 2003 to 2006 revealed that 94% of these patients were treated with intravenous BPs (primarily Zol and pamidronate), and 85% had multiple myeloma or metastatic breast cancer.(15,16) The high incidence of BRONJ in the group of cancer patients who are also taking multiple immunosuppressive drugs suggests that an altered immunologic status may contribute an associated risk factor aside from the apparent large cumulative dose of BP. We explore whether alteration of host immunity contributes to BRONJ disease incidence and activity. Since Treg suppression can contribute to immunotolerance, we postulate that treatment with anti-CD25 antibody (CD25Ab) may aggravate BRONJ-like disease in wild-type C57BL/6J mice treated with Zol and Dex. As expected, we observed a higher incidence, 67% versus 50%, of BRONJ-like lesions, manifested as an open extraction socket with areas of exposed bone and no mucosal coverage, in mice treated with CD25Ab at 2 weeks after extraction compared with Zol- and Dex-treated C57BL/6J mice (Fig. 2*A, B*). Furthermore, to confirm Treg function in BRONJ-like disease, thymus-derived Tregs were systemically infused in BRONJ mice at 2 days after tooth extraction. Restoration of Treg function by adaptive transfer of isolated syngeneic Tregs prevented development of BRONJ-like lesions and resulted in normal socket healing, shown as excellent soft tissue healing clinically (Fig. 2*A, B*), complete epithelial coverage and bone regeneration histologically (Fig. 2*C, D*), and marked bone filling at the extracted alveolar socket radiographically (Fig. 2*C*). This evidence suggests a potential role of immunotherapy in restoring Treg function as a novel approach to treat or prevent BRONJ-like lesions in mice.

It has been recognized that MSCs are capable of unique immunomodulating properties(25,26) and function to harness the inflammatory response.(27,28) Here we test the feasibility of immunotherapy using an MSC-based approach to treat BRONJ. At 2 weeks after tooth extraction, BRONJ-like wild-type C57BL/6J mice receiving MSC transplantation healed with complete soft tissue and bone regeneration at the extracted alveolar socket (Fig. 2*A–D*). Representative histologic images of extracted socket showed complete epithelial coverage (Fig. 2*C*, *open arrow*) and newly formed bone at the extraction site (mucosa and socket, H&E) compared with an open socket with no epithelial coverage (*blue dot line*) and the presence of necrotic bone and localized inflammatory infiltrates in CD25Ab-treated BRONJ-like mice (Fig. 2*C, D*). Accordingly, µCT analysis showed a markedly delayed bone healing at the extraction socket of the CD25Ab-treated group. In both Treg- and MSC-treated groups, adequate bone regeneration at the alveolar sockets was observed at 2 weeks after extraction (Fig. 2*C, D*).

This experimental evidence suggests that cell-based immunotherapy using Tregs or MSCs is a promising therapeutic strategy to prevent and treat human BRONJ-like lesions in wild-type mice. Therefore, we hypothesize that alteration in the immune homeostasis, driven by a suppressed level of Tregs, may render mice more susceptible to BRONJ development when undergoing treatment with high-dose intravenous BPs and Dex.

### Tregs and Treg/Th17 ratio were suppressed in BRONJ-like disease model

We examine whether Zol regulates Treg function. Increasing evidence, both in vitro and in vivo, supports that nitrogen-containing BPs are capable of modulating both innate and adaptive immune responses.(29–31) Immunomodulation by BP treatment can result in either immunosuppression or a generalized enhanced immune response that subsequently promotes the development of BRONJ. We evaluate the effect of Zol treatment by itself or in conjunction with Dex on the levels of Tregs and Th17 in the peripheral blood of mice at 2 weeks after tooth extraction. Zol-only- and combination of Zol- and Dex-treated mice showed a significantly decreased level of CD4^+^CD25^+^Foxp3^+^ cells (Tregs) in the peripheral blood compared with untreated control with and without tooth extraction (Fig. 3*A*). Dex treatment increased Treg level in peripheral blood compared with all control groups (Fig. 3*A*). However, tooth extraction and Dex and Zol treatment suppressed IL17^+^IFNγ^−^ cells at 2 weeks after tooth extraction (Fig. 3*B*). On the other hand, Zol and Dex treatment significantly increased the level of CD4^+^IL17^+^IFNγ^−^ cells (Th17) (Fig. 3*B*). Consequently, the ratio of CD4^+^CD25^+^Foxp3^+^ and CD4^+^IL17^+^IFNγ^−^ cells was markedly decreased in the Zol- and Dex-treated group compared with other groups (Fig. 3*C*). These findings suggest that combination of Zol and Dex treatment is capable of suppressing the Treg/Th17 ratio in the peripheral blood at 2 weeks after tooth extraction, suggesting that deficiency in Treg level and Treg/Th17 ratio potentially may correlate with BRONJ-like disease in mice.

Since surgical insult or tooth extraction has been correlated with an increased risk for BRONJ in humans, we next determine whether this factor affects Treg and Th17 immune components at 2 weeks after tooth extraction. We found that tooth extraction increased levels of CD4^+^CD25^+^Foxp3^+^ and CD4^+^IL17^+^IFNγ^−^ cells in Zol- and Dex-treated group (Supplemental Fig. 2*A, B*). Consistent with these findings, tooth extraction caused a mild suppression of the Treg/Th17 ratio in Zol- and Dex-treated mice (Supplemental Fig. 2*C*).

### Cell-based immunotherapy rescued Treg deficiency and altered serum cytokine levels in Zol- and Dex-treated mice

We next examine whether therapeutic infusion with MSCs and Tregs (CD4^+^CD25^+^ T cells derived from thymus) alters host immune functions, particularly Treg and Th17 cells, in BRONJ-like wild-type C57BL/6J mice. First, we confirmed that treatment with CD25Ab targeting CD4^+^CD25^+^ cells resulted in further suppression of CD4^+^CD25^+^Foxp3^+^ Treg level in the peripheral blood of CD25Ab-treated BRONJ-like mice compared with Zol- and Dex-treated mice and tooth extraction control at 2 weeks after tooth extraction (Fig. 3*D*). Immunotherapy with Tregs and MSCs recovered the level of CD4^+^CD25^+^Foxp3^+^ cells (Fig. 3*D*) and reciprocally suppressed CD4^+^IL17^+^IFNγ^−^ cells in Zol- and Dex-treated mice (Fig. 3*E*). As a result, the ratio of CD4^+^CD25^+^Foxp3^+^ Tregs and CD4^+^IL17^+^IFNγ^−^ Th17s in the peripheral blood was significantly enhanced in both Treg- and MSC-treated mice compared with Zol- and Dex-treated mice (Fig. 3*F*). These treatment outcomes provide the first evidence that immunotherapy with MSCs and Tregs can reverse Treg deficiency in BRONJ-like mice, correlating with reversal of BRONJ-like lesions in mice.

We next explore the serum cytokine profile of mice in response to cell-based infusion therapies. Systemically, we observed a reduced serum IL-10 level in Zol- and Dex-treated and CD25Ab-treated mice that was recovered with Treg and MSC treatment compared with the tooth extraction group at 7 weeks after extraction (Fig. 4*A*). Serum IL-6 level, a proinflammatory cytokine, was activated in Zol- and Dex-treated and CD25Ab-treated mice and was suppressed following Treg and MSC infusions (Fig. 4*B*). Likewise, serum IL-17, another proinflammatory cytokine, was upregulated in Zol- and Dex-treated and CD25Ab-treated groups and was restored to basal level after Treg and MSC infusions (Fig. 4*C*). Consistence with the elevated level of proinflammatory cytokines, we observed a mild increase in C-reactive protein (CRP), a general marker of the inflammatory response, in both Zol- and Dex-treated and CD25Ab-treated groups compared with other groups (Fig. 4*D*). We also observed a suppressed level of transforming growth factor β1 (TGF-β1) in peripheral blood following Zol and Dex and CD25Ab treatments; the level of serum TGF-β1 was partially restored in the Treg-treated group and slightly enhanced in the MSC-treated group (Fig. 4*E*).

### Altered immune homeostasis constitutes an increased risk of BRONJ disease

**Fig. 5:**
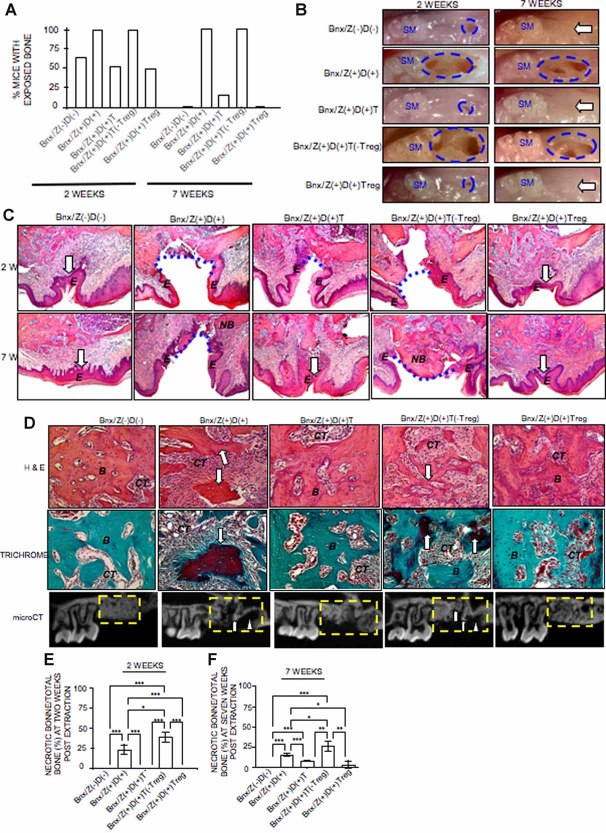
Development of BRONJ-like lesions in immunocompromised mice. (*A*) Incidence of BRONJ-like lesion, manifested as an unhealed open socket with an area of exposed bone and no mucosal coverage, at the extraction site in Beige *nu/nu* Xid (III) (*Bnx*) mice treated with Zol (Z) alone (*Bnx*/Z^+^D^−^) or in combination with Dex (D) (*Bnx*/Z^+^D^+^) for 3 and 8 weeks, whereby mice with no treatment (*Bnx*/Z^−^D^−^) served as controls. To test adaptive transfer of pan-T and Treg cells in the treatment of BRONJ-like lesion, three experimental groups were included: *Bnx*/Z^+^D^+^ with intravenous infusion of either pan-T cells (*Bnx*/Z^+^D^+^T) or T cells with depleted Tregs [*Bnx*/Z^+^D^+^T (–Treg)] or purified Tregs (*Bnx*/Z^+^D^+^Treg). T cell infusion was performed 2 days after tooth extraction. (*B*) Representative gross clinical appearance of gingival mucosa at the extraction sites at 2 and 7 weeks after tooth extraction. Blue circles represent images of the apparent mucosal disruption with exposed bone at the extracted site adjacent to the second molar (SM), whereas open arrows point to the healed gingival mucosa. (*C*) Histologic images of extraction sockets showing open socket without epithelial lining (*blue dot line*) and healed gingival mucosa with complete epithelial coverage (*open arrow*). NB = necrotic bone. (*D*) H&E (*upper panel*) and trichrome (*middle panel*) staining of extraction socket areas displaying newly formed bone (B), connective tissues (CT), and necrotic bone (NB) at 7 weeks after tooth extraction. Open arrows point to the necrotic bones. µCT analysis (*lower panel*) showed necrotic bone (*open arrow*) and reduced bone formation (*triangle*) in *Bnx*/Z^+^D^+^ and *Bnx*/Z^+^D^+^T (–Treg) groups. (*E*, *F*) Quantification of necrotic bone areas in *Bnx*/Z^+^D^+^ and mice treated with systemic infusion of different T cells at 2 and 7 weeks after tooth extraction. *p < .05; **p < .01; ***p < .005. Bars indicate SD. The results are representative of three independent experiments.

To further confirm the role of Treg-associated immunomodulation in BRONJ disease, we selected beige *nu/nu* Xid (III) immunocompromised (*Bnx*) mice as a study model. At both 2 and 7 weeks after tooth extraction, 100% of Zol- and Dex-treated *Bnx* mice displayed open sockets with exposed alveolar bone and no epithelial coverage versus 67% and none in the untreated control mice, respectively (Fig. 5*A, B*). Histologic analyses confirmed the lack of epithelial coverage and the presence of necrotic bone islands or sequestra in Zol- and Dex-treated *Bnx* mice up to 7 weeks after extraction (Fig. 5*C, D*). In untreated control immunocompromised mice, an intact epithelial lining was observed in the alveolar socket of 30% to 33% of mice at 2 weeks, followed by complete soft tissue granulation and mucosal coverage of the extracted socket in all mice at 7 weeks (Fig. 5*B, C*). Bone regeneration was observed in the extracted alveolar socket characterized by newly formed woven bone at 2 weeks (Fig. 5*C*) and lamellar bone at 7 weeks (Fig. 5*D*), leading to restoration of the voided socket. In Zol- and Dex-treated *Bnx* mice, an empty socket with a local opaque intrabony bone and maxillary sinus floor fracture was observed (Fig. 5*D*). These opaque bone islands appeared to correlate with the necrotic bones or sequestra exposed in the open sockets (Fig. 5*D*).

To verify that immunologic dysfunction contributes to the development of BRONJ, we performed adaptive immune transfer using Pan-T-lymphocytes prior to surgical extraction in Zol- and Dex-treated *Bnx* mice. Interestingly, we observed a decreased incidence of BRONJ from 100% to 50% at 2 weeks and to 14.3% at 7 weeks after extraction (Fig. 5*A*). Clinical examination, confirmed by histologic study, reveals a partly epithelial coverage at 2 weeks (Fig. 5*B, C*) and complete closure at 7 weeks (Fig. 5*B, C*). Histologic and radiographic studies using H&E staining and µCT, respectively, revealed moderate bone regeneration in pan-T cell–treated *Bnx* mice compared with the empty socket in Zol- and Dex-treated *Bnx* mice with BRONJ-like lesions (Fig. 5*D*).

We next determine which component of the immune system plays a role in preventing BRONJ development in Zol- and Dex-treated *Bnx* mice using adaptive immune transfer with purified CD4^+^CD25^+^ thymus-derived T cells (Tregs) or CD4^+^CD25^+^-depleted spleen-derived T cells (depleted Tregs). Immunotherapy with CD4^+^CD25^+^ Tregs, not depleted Tregs, reduced BRONJ-like clinical manifestations from 100% to 50% at 2 weeks after extraction and completely suppressed BRONJ development at 7 weeks (Fig. 5*A–C*). Histologic examination confirmed the preceding clinical findings, showing a similar course of epithelial healing and bone formation at 2 and 7 weeks after tooth extraction compared with untreated control mice (Fig. 5*C, D*). Likewise, µCT findings confirmed remarkable bone filling at the extracted socket, similar to normal bone regeneration in control mice (Fig. 5*D*).

Bone necrosis was quantified as the average value of the percentage of necrotic bone over the total area of bone (Fig. 5*E, F*). Zol and Dex treatment resulted in marked bone necrosis at 2 and 7 weeks after tooth extraction (Fig. 5*E, F*). Convincingly, immunotherapy with pan-T cells and Tregs, but not T cells with Treg depletion, suppressed BRONJ-like lesions at extracted socket sites of all Treg-treated mice and 90% of pan-T-treated mice (Fig. 5*E, F*). At the bone cellular level, we observed an overall suppression of osteoclasts in Zol- and Dex-treated *Bnx* mice with BRONJ-like lesions (Supplemental Fig. 1*B*) that was restored by infusion therapy with Tregs.

## Discussion

The highest incidence of BRONJ is reported in oncology patients receiving immunosuppressive therapy. A systematic review of reported cases of BRONJ from 2003 to 2006 revealed that 94% of these patients were treated with intravenous BPs (primarily pamidronate and Zol), and 85% were diagnosed with multiple myeloma or metastatic breast cancer.(15,16) It is recognized that 60% of cases occurred following a tooth extraction or other dentoalveolar surgery, and the remaining cases occurred spontaneously. Several important predisposing factors for BRONJ have been identified, including the type and total dose of BP, history of trauma, dental surgery, impaired immunity, and development of dental infection. The estimated incidence of BRONJ in patients receiving intravenous BPs for malignant diseases ranges from 0.8% to 12%,(18) an incidence that is 900 times higher than that of general osteoporosis patients.(19) Despite these identified clinical correlates with the incidence of BRONJ, no evidence-based study has established a true direct causal effect. Therefore, the establishment of a BRONJ-like animal model would be a critical milestone for defining the pathogenesis of the disease, its etiology, and potential risk factors. Recent studies in rats showed that administration of Zol acid and Dex preceding a dental extraction resulted in bone and soft tissue changes that resembled those noted in human BRONJ.(32) The doses for oncologic indications of BP are 10 to 12 times higher than those used in osteoporosis(3,17) and usually in combination with an immunosuppressive agent such as corticosteroids.(15,16) In this study, we adapt a similar treatment regimen for cancer patients to induce BRONJ in mice. Recognizing that the cumulative high dose of BP has an effect on the progressive course of BRONJ and the relatively shorter lifespan of the mouse compared with human, we titrated Zol dose to achieve a concentration that produced a repeatable clinical effect in the generation of our murine BRONJ-like model without renal toxicity (data not shown). The use of high-dose Dex in conjunction with BPs has been adapted to mice using the equivalent treatment regimen for advanced multiple myeloma.(15) The addition of Dex in conjunction with BP treatment significantly increased the incidence of BRONJ in wild-type mice compared with Zol alone, suggesting that immunosuppressive therapy may render mice more susceptible to BRONJ lesions. This observation is in accordance with human epidemiologic studies showing a much higher incidence of BRONJ in cancer patients receiving immunosuppresive therapy.(15,16)

We showed that BRONJ-like lesions exhibit similar characteristics to human disease involving mucosal ulceration or open sockets, osseous sclerosis, exposed necrotic bone and sequestra, and radiopaque alveolar bone in the jaw, as demonstrated by µCT and histologic studies. The alveolar bone of BRONJ-like lesion failed to remodel up to 7 weeks after extraction and in some cases up to 12 weeks (Supplemental Fig. 3), resulting in accumulation of necrotic bone. The clinical manifestations of BRONJ, specifically open sockets with exposed necrotic bone and no mucosal lining, persisted beyond the 2- to 3-week normal course of healing seen in nontreated control mice and therefore met the current diagnosis criteria of human BRONJ.(5,17,24) The hallmark of persistent presence of BRONJ-like clinical, radiographic, and histologic features at the extraction site supports our model of BRONJ-like lesion in mice and allows accurate delineation of the pathophysiologic mechanisms of the disease, leading to the future development of new clinical and laboratory endpoints for accurate diagnosis and prediction of BRONJ in humans.

Since most cancer patients are on multiple immunosuppressant drugs, including Dex, and chemotherapeutic agents and therefore experience some degree of impaired immunity, it is postulated that immunosuppression may contribute to an increased susceptibility to BRONJ. Recently, increasing evidence, both in vitro and in vivo, supports the idea that BPs are able to regulate the immune system by modulating both innate and adaptive immune responses(20,24,29) and impairing monocyte/macrophage and dendritic cell maturation and function.(30,31) Immunomodulation by BP treatment can result in either immunosuppression or generalized enhanced immune responses(19) that subsequently may promote the development of BRONJ. More important, in our preliminary studies, attempts to develop BRONJ-like animal models using different strains of mice revealed that nearly 100% of immunocompromised mice versus 33% of wild-type C57BL/6J mice treated with oncologic doses of Zol and Dex developed BRONJ-like pathologic lesions at 7 weeks after tooth extraction. Furthermore, studies using adaptive immune transfer with pan-T-lymphocytes prior to surgical tooth extraction in Zol- and Dex-treated immunocompromised mice resulted in a reduced incidence of BRONJ from 100% to 50% at 2 weeks and to 14.3% at 8 weeks after extraction. This effect was not observed when Tregs were depleted from the pan-T-lymphocyte pool. This compelling evidence confirms our hypothesis that altered immune homeostasis, specifically a lack of functional T cells in immunocompromised mice, renders these mice more susceptible to BRONJ-like lesions.

To further delineate the specific immune components contributing to BRONJ susceptibility in our model, we explore the role of Tregs, a subpopulation of T cells capable of suppressing various immune responses and thereby regulating immune homeostasis and tolerance to antigens.(33,34) Tregs have been shown in vitro and in vivo to suppress IL-17-producing T-helper cells, Th17s, and their reciprocal relationship is modulated by the acute inflammatory protein IL-6.(35) Th17 cells are important players in the etiopathogenesis of several inflammatory and autoimmune diseases, including rheumatoid arthritis (RA), multiple sclerosis (MS), systemic lupus erythematosus (SLE), inflammatory bowel disease (IBD), psoriasis,(36–39) and chronic periodontal lesions.(30–42) There exist two major subtypes of CD4^+^CD25^+^ Tregs, the naturally occurring CD4^+^CD25^+^Foxp3^+^ Tregs (nTregs) that originate in the thymus and the induced CD4^+^CD25^+^Foxp3^+^ Tregs (iTregs) that are generated in the periphery. In our study, we observed that systemic infusion of Tregs completely prevented the development of BRONJ-like lesions at 7 weeks after extraction. To further confirm our hypothesis, we treated BRONJ-like mice with anti-CD25Ab and observed exacerbation of BRONJ-like disease, shown as an increased incidence of open socket with exposed bone from 50% to 66% at 2 weeks. Mechanistically, we demonstrated that treatment with Zol/Dex suppressed both Tregs and the Treg/Th17 ratio in peripheral blood.

Recently, the immunomodulatory and anti-inflammatory effects of MSCs have been reported in a variety of animal models, and it is speculated that they play a potential role in certain human diseases. Interestingly, our data showed that treatment with systemic infusion of MSCs resulted in a marked suppression of the Zol- and Dex-induced increase in Th17 cells in peripheral blood and restoration of Treg levels in the treated BRONJ-like mice. At 2 weeks after extraction, BRONJ-like wild-type C57BL/6J mice receiving MSC infusions showed complete mucosal healing and bone regeneration at the extracted alveolar socket. To our knowledge, these promising treatment outcomes provide the first evidence that immunotherapy with MSCs and Tregs can reverse Treg deficiency in BRONJ-like mice and is capable of preventing and curing BRONJ-like lesions in mice.

In our model, we observed BRONJ-like lesions only at the site induced by dental extraction, making BRONJ a condition that affected only the oral cavity. However, it is not surprising that the underlying Zol- and Dex-induced necrotic bone presumably could occur at other skeletal sites if they were traumatized. Further studies are in progress to determine the potential systemic effect of Zol. We recognized that several other mechanisms, including osteoblast function, angiogenesis, dental infection, and conditions linked to altered immune homeostasis, potentially contribute to the pathogenesis of BRONJ. Even though bacterial colonization has been reported in BRONJ, as shown at the bottom of the extraction socket (Supplemental Fig. 4), it is still unknown whether the infection arises initially in the bone or soft tissue or whether it primarily plays a causative role in the pathogenesis of BRONJ.(43,44) In this study, we observed that necrotic bone was often found adjacent to area of intense local inflammatory infiltrates, suggesting an association between inflammation, microbial flora, and tissue degeneration/necrosis in BRONJ-like disease. The underlying host immunocompromise in conjunction with other risk factors unique for the oral cavity, specifically the open wound and the oral microbial flora, may contribute to the high incidence of BRONJ in patients undergoing dental surgeries. Further studies are needed to verify whether infection constitutes a risk factor in BRONJ development.

In summary, despite the potential risk of developing BRONJ in a subpopulation of patients, BP has greatly benefited a large number of patients suffering from skeletal complications related to bone metabolism and neoplasms. Our animal models will allow the direct evaluation of clinically identified risk factors in the development of BRONJ and the garnering of evidence-based guidance leading to the development of preventive, diagnostic, and therapeutic tools in the management of human BRONJ. More important, cell-based immunotherapy using systemic MSCs and Tregs potentially can offer a safe and effective novel therapeutic modality in preventing the development of BRONJ disease in vulnerable cancer patients who have to undergo BP treatment for their cancer, as well as the larger group of patients who are on oral BPs for bone-related metabolism conditions.
