# Supplementary material for: Cell-Based Immunotherapy With Mesenchymal Stem Cells Cures Bisphosphonate-Related Osteonecrosis of the Jaw–like Disease in Mice
Source: J Bone Miner Res. 2010 Jan 29;25(7):1668–79. doi: 10.1002/jbmr.37 (PMC3154005; doi:10.1002/jbmr.37)
Supplement: Supplementary file 1 [file jbmr0025-1668-SD1.ppt]

## Slide 1
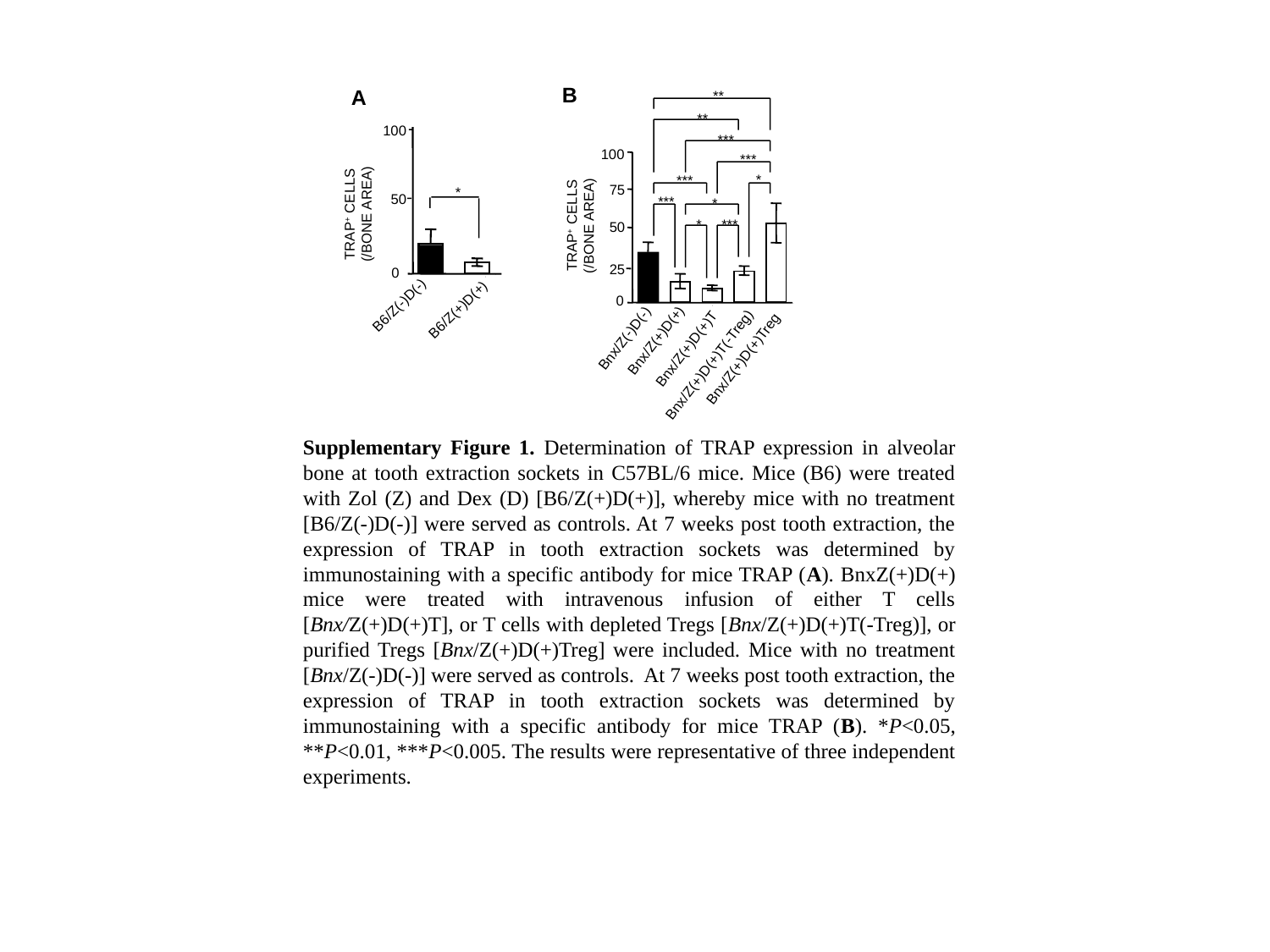

B
**
**
***
***
100
*
***
75
***
*
TRAP+ CELLS
(/BONE AREA)
*
***
50
25
0
1
Bnx/Z(+)D(+)
Bnx/Z(-)D(-)
Bnx/Z(+)D(+)Treg
Bnx/Z(+)D(+)T
A
100
*
50
TRAP+ CELLS
(/BONE AREA)
0
1
B6/Z(-)D(-)
B6/Z(+)D(+)
Bnx/Z(+)D(+)T(-Treg)
Supplementary Figure 1. Determination of TRAP expression in alveolar bone at tooth extraction sockets in C57BL/6 mice. Mice (B6) were treated with Zol (Z) and Dex (D) [B6/Z(+)D(+)], whereby mice with no treatment [B6/Z(-)D(-)] were served as controls. At 7 weeks post tooth extraction, the expression of TRAP in tooth extraction sockets was determined by immunostaining with a specific antibody for mice TRAP (A). BnxZ(+)D(+) mice were treated with intravenous infusion of either T cells [Bnx/Z(+)D(+)T], or T cells with depleted Tregs [Bnx/Z(+)D(+)T(-Treg)], or purified Tregs [Bnx/Z(+)D(+)Treg] were included. Mice with no treatment [Bnx/Z(-)D(-)] were served as controls. At 7 weeks post tooth extraction, the expression of TRAP in tooth extraction sockets was determined by immunostaining with a specific antibody for mice TRAP (B). *P<0.05, **P<0.01, ***P<0.005. The results were representative of three independent experiments.

## Slide 2
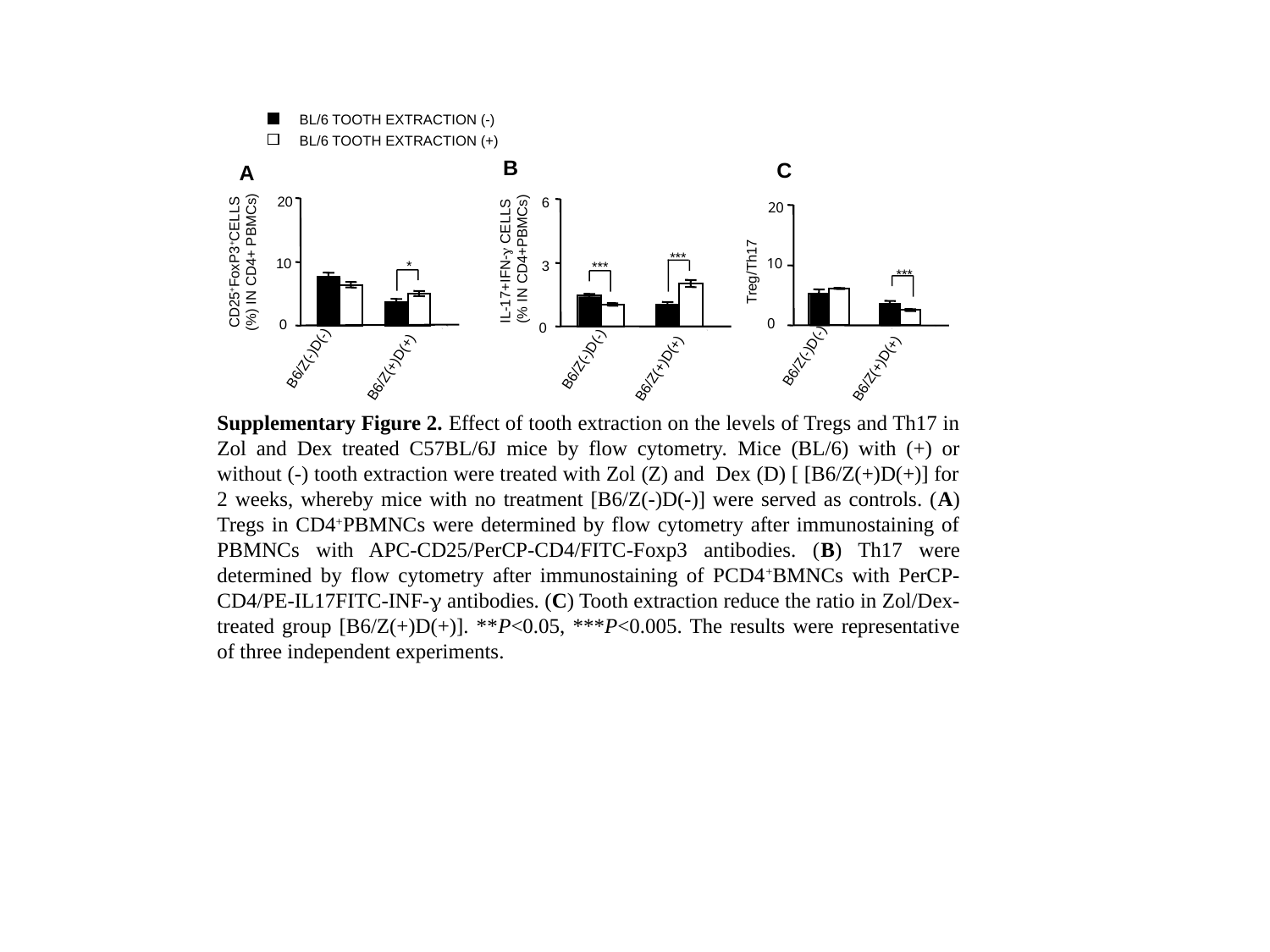

BL/6 TOOTH EXTRACTION (-)
A
20
CD25+FoxP3+CELLS
(%) IN CD4+ PBMCs)
*
10
0
1
B6/Z(-)D(-)
B6/Z(+)D(+)
BL/6 TOOTH EXTRACTION (+)
B
6
IL-17+IFN-γ CELLS
(% IN CD4+PBMCs)
***
***
3
0
B6/Z(-)D(-)
C
20
10
Treg/Th17
***
0
1
1
B6/Z(-)D(-)
B6/Z(+)D(+)
B6/Z(+)D(+)
Supplementary Figure 2. Effect of tooth extraction on the levels of Tregs and Th17 in Zol and Dex treated C57BL/6J mice by flow cytometry. Mice (BL/6) with (+) or without (-) tooth extraction were treated with Zol (Z) and Dex (D) [ [B6/Z(+)D(+)] for 2 weeks, whereby mice with no treatment [B6/Z(-)D(-)] were served as controls. (A) Tregs in CD4+PBMNCs were determined by flow cytometry after immunostaining of PBMNCs with APC-CD25/PerCP-CD4/FITC-Foxp3 antibodies. (B) Th17 were determined by flow cytometry after immunostaining of PCD4+BMNCs with PerCP-CD4/PE-IL17FITC-INF-γ antibodies. (C) Tooth extraction reduce the ratio in Zol/Dex-treated group [B6/Z(+)D(+)]. **P<0.05, ***P<0.005. The results were representative of three independent experiments.

## Slide 3
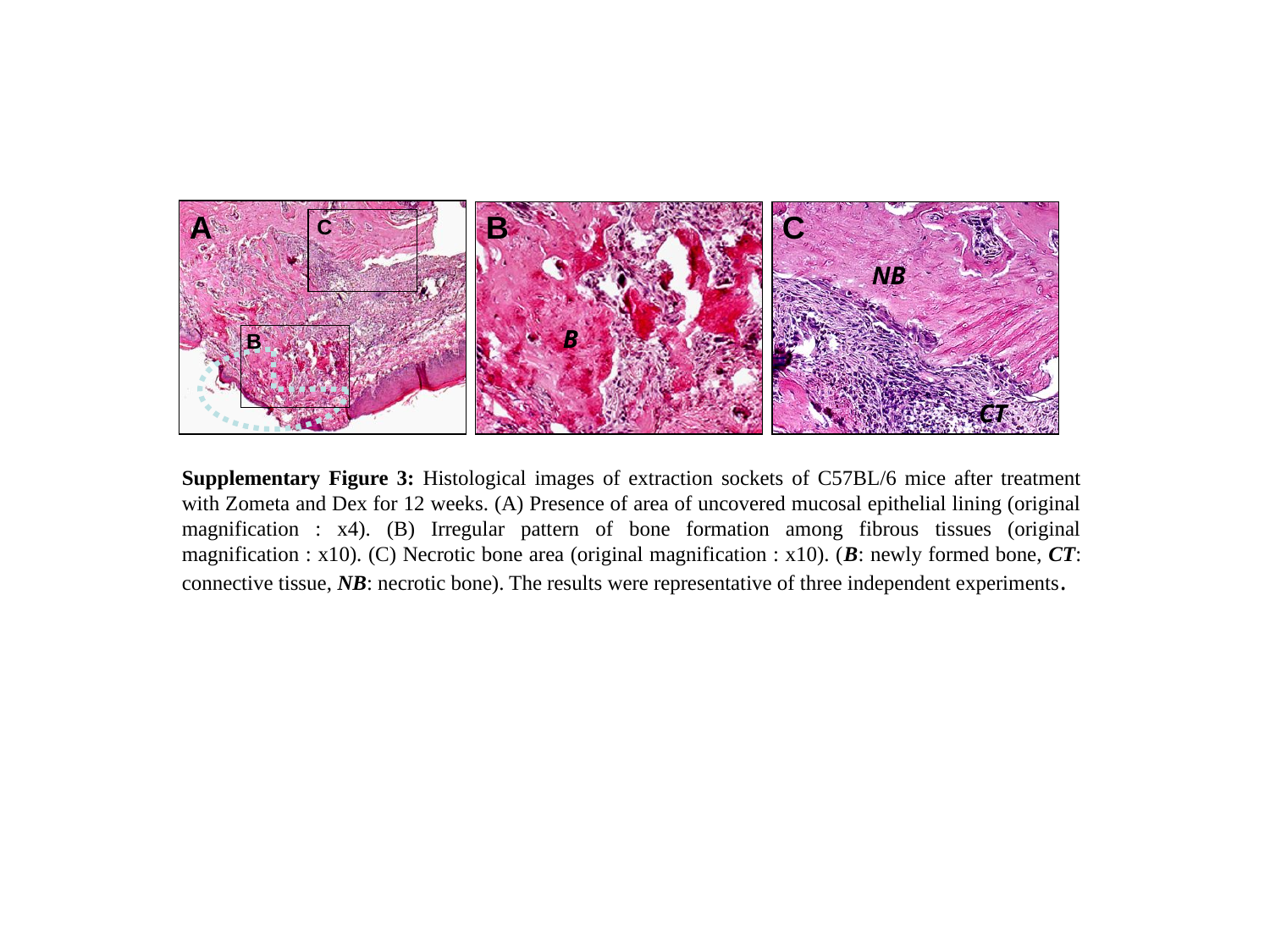

A
B
C
C
NB
B
B
CT
Supplementary Figure 3: Histological images of extraction sockets of C57BL/6 mice after treatment with Zometa and Dex for 12 weeks. (A) Presence of area of uncovered mucosal epithelial lining (original magnification : x4). (B) Irregular pattern of bone formation among fibrous tissues (original magnification : x10). (C) Necrotic bone area (original magnification : x10). (B: newly formed bone, CT: connective tissue, NB: necrotic bone). The results were representative of three independent experiments.

## Slide 4
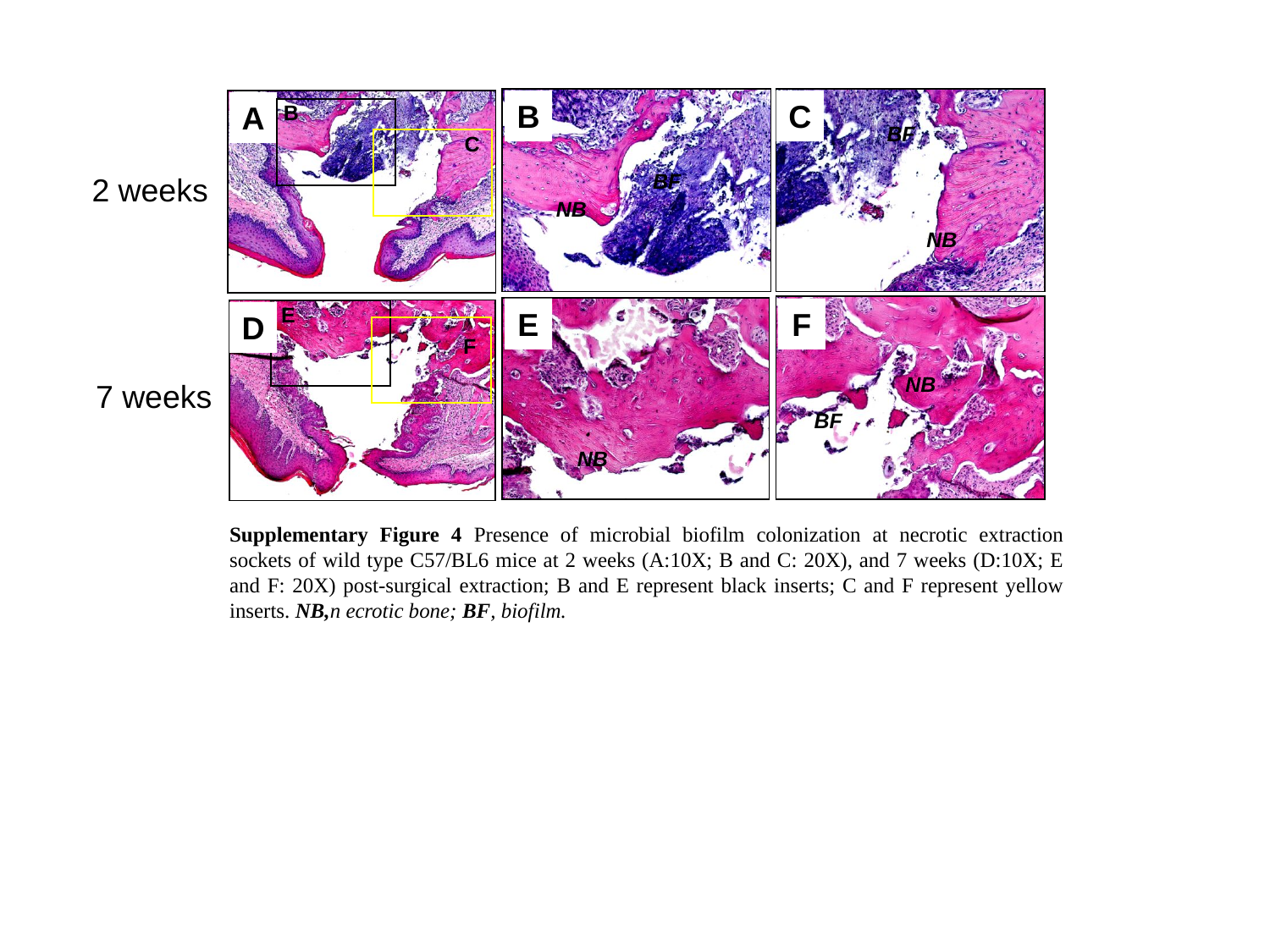

B
C
A
B
BF
C
BF
2 weeks
NB
NB
E
E
F
D
F
NB
7 weeks
BF
NB
Supplementary Figure 4 Presence of microbial biofilm colonization at necrotic extraction sockets of wild type C57/BL6 mice at 2 weeks (A:10X; B and C: 20X), and 7 weeks (D:10X; E and F: 20X) post-surgical extraction; B and E represent black inserts; C and F represent yellow inserts. NB,n ecrotic bone; BF, biofilm.
