# Supplementary material for: Cell-Based Immunotherapy With Mesenchymal Stem Cells Cures Bisphosphonate-Related Osteonecrosis of the Jaw–like Disease in Mice
Source: J Bone Miner Res. 2010 Jan 29;25(7):1668–79. doi: 10.1002/jbmr.37 (PMC3154005; doi:10.1002/jbmr.37)
Supplement: Supplementary file 2 [file jbmr0025-1668-SD2.doc]

**Supplementary Information**

**Cell-based Immunotherapy with Mesenchymal Stem Cells Cures Bisphosphonate-Related Osteonecrosis of the Jaw-like Disease in Mice**

Takashi Kikuiri^1*^, Insoo Kim^1*^, Takyoshi Yamaza^1^, Kentaro Akiyama^1^, Qunzhou Zhang^1^, Yunsheng Li^2^, Chider Chen^1^, WanJun Chen^3^, Songlin Wang^2^, Anh D. Le^1^, Songtao Shi^1^

^1^Center for Craniofacial Molecular Biology, University of Southern California, School of Dentistry,

Los Angeles, California, 90033

^2^Salivary Gland Disease Center and the Molecular Laboratory for Gene Therapy & Tooth Regeneration, Capital Medical University School of Stomatology, Beijing 100050, China

^3^National Institute of Dental and Craniofacial Research, National Institutes of Health, Bethesda, Maryland 20892, USA

**MATERIALS AND METHODS**

*Animals*

Mice were individually identified by ear punching and housed two-five animals per experimental group in each cage with appropriate label. They were housed under temperature- (72 °F ± 3°) and air- (50 ± 20% relative humidity)-controlled condition, and given a standard diet and water ad libitum throughout this study. Animals were acclimatized prior to the study and animals with signs of distress were rejected from the study inclusion.

*Antibodies*

PerCP-conjugated anti-mouse CD4, FITC-conjugated anti-mouse CD8a, APC-conjugated anti-mouse CD25, and PE-conjugated anti-mouse IL17, were purchased from BD Bioscience. PE-conjugated anti-mouse Foxp3 was obtained from eBioscience. PE-conjugated anti-mouse CD25 and microbeads conjugated anti-PE antibodies were from MiltenyiBiotec. Anti-CD25 monoclonal rat IgG1 antibody for neutralization assay *in vivo* was produced by hybridoma PC61 according to previous study (1,2).

*Purification of PanTs and Tregs and depletion of Tregs from PanTs*

PanTs were negatively depleted from single cell suspension derived from splenocytes of C57BL/6J mice by means of MidiMACS™ (Miltenyi Biotec) and MACS® separator columns (Miltenyi Biotec) using mouse PanT cell isolation kit (Miltenyi Biotec) following manufacturer’s instruction. Tregs were positively selected from single cell suspension of thymocytes of C57BL/6J mice using mouse CD4^+^CD25^+^ regulatory T cell isolation kit (Miltenyi Biotec). To deplete Tregs population from PanTs, purified-PanT cells were incubated with PE-conjugated anti-mouse CD25 antibody (Miltenyi Biotec), followed by the reaction with microbeads conjugated anti-PE antibody (Miltenyi Biotec), and negatively separated using MidiMACS™ and MACS® separator columns**.**

*Adaptive transfer of T-lymphocytes into immunocompromised mice*

After isolation, the cells were washed with cold saline for three times and kept on ice until the injection. Each T-lymphocyte population (1x10^6^/100 μl saline/mouse) was intravenously infused into immunocompromised mice via tail vein two days after tooth extraction. Mice injected with saline (100 μl /10 g body weight) were used as controls.

*Mouse BMMSC isolation and culture*

Bone marrow cells were flashed out from bone marrow cavity of femurs and tibias with PBS containing heat-inactivated 3% fetal bovine serum (FBS; Equitech-Bio) and antibiotics (100 U/ml penicillin and 100 µg/ml streptomycin; Biofluids). All nuclear cells (ANCs) were seeded at 15 x 10^6^ into 100 mm culture dishes (Corning) and initially incubated for 3 hours under 37^o^C at 5% CO_2_ condition. To eliminate the non-adherent cells, the cultures were washed with PBS twice. The attached cells were cultured for 16 days. Colonies-forming attached cells were passaged once prior to experiments. The BMMSCs were cultured with alpha minimum essential medium (α-MEM) (Invitrogen) supplemented with 20% FBS, 2 mM L-glutamine (Invitrogen), 55 µM 2-mercaptoethanol (Invitrogen) and antibiotics (100 U/ml penicillin and 100 µg/ml streptomycin).

*Mouse biological sample preparation*

At each time point, maxilla and peripheral blood (PB) were harvested. PB was collected into Microtainer tubes® (BD Bioscience) from the retro-orbital plexus of the mice. The sera were separated according to the manufacturer’s protocol, and kept at -20°C until used. Some blood samples were collected in Microtainer tubes® with EDTA (BD Bioscience) to use for flow cytometric analysis. Intact en-bloc maxilla covering oral mucosa was fixed with 4% paraformaldehyde (PFA) in phosphate buffered saline (PBS), pH 7.4, at 4°C. Intact maxillary specimens were used for μCT analysis. Other maxilla specimens were decalcified with 5% ethyrendiaminotetraacetic acid (EDTA) in PBS, pH 7.4, at 4°C. Demineralized maxillary samples were dehydrated, cleaned with xylene, and embedded in paraffin. Single cell suspension was isolated from unfixed spleen for further experiments grown at sub-confluent condition and collected with TrypLE™ Express (Invitrogen). Cell pellets were washed with cold saline for three times and kept on ice before the injection. Under deep anesthesia, MSC suspension (1x10^6^/100 μl saline/mouse) was intravenously infused into BL/6 mice through tail vein two days after tooth extraction. Saline-injected mice (100 μl /mouse) were used as controls.

*Clinical evaluation of extracted tooth socket*

Oral mucosa covering the extracted socket in the maxilla was photographed at the same magnification. The absence of mucosal coverage and presence of exposed bone was gently probed using a fine explorer. The incidence of BRONJ-like lesion was determined as % of mice showing open socket with area of exposed bone and no mucosal coverage to total mice in each experimental group at 2 and 7 weeks post extraction.

**Micro-CT analysis.** Intact and unprocessed maxilla specimens were harvested at the indicated time points. The maxilla was scanned using a μCT machine (Siemens Inveon CT) and multi sliced CT sections (5-10 microns thickness), specifically at the tooth extraction regions corresponding to the first molars, were collected, and two dimensional images were analyzed using software from Siemens Medical Solutions USA, Inc.

*Histological analysis*

Tissues were fixed with 4% PFA in PBS, pH 7.4, overnight at 4°C, and decalcified with 5% EDTA in PBS, pH 7.4 for 10 days at 4°C. Bone samples were dehydrated with a graduate series of ethanol, cleaned with xylene, and immersed in paraffin. The samples were embedded in paraffin and cut into 8-μm-thick sections. The sections were deparaffinized, rehydrated and used for H&E staining and further histochemical staining.

*Trichrome staining*

De-paraffinized sections were incubated hematoxylene solution, followed by Gomori’s trichrome staining solution.

*TRAP staining*

TRAP staining was performed as described (1). Briefly, de-waxed sections were treated with 50% acetone and 50% ethanol solution for 10 min, incubated with TRAP-staining solutions for 10 min at 37^o^C. TRAP-staining solutions were a mixure of 1.6% naphthol AS-BI phosphate in N, N-dimethylformamide and 0.14% fast red-violet LB diazonium salt, 0.097% tartaric acid and 0.04% MgCl2 in 0.2 M sodium acetate buffer (pH 5.0). The sections were counterstained with toluidine blue. All regents for TRAP staining were purchased from Sigma.

*Histomorphometry*

The necrotic and total bone areas were analyzed using H&E stained slides. To quantify osteoclast activity in the bones, the number of mature osteoclasts was determined as TRAP positive cells attached on the bone surface. The number of osteoclast cells and the bone area were measured from five to seven representative images of each sample using an NIH Image-J. The data were the average means in each experimental group. The results were shown indicated percentage.

*Flow cytometric analysis*

Mononuclear cells (MNCs) were isolated from PB treated with ACK lysing buffer (Lonza) to remove red blood cells. For Treg staining, PBMNCs (100-200 x 10^3^ per subject) were stained with PerCP-conjugated anti-CD4, FITC-conjugated anti-CD8a and APC-conjugated anti-CD25 antibodies for 30 minutes under the shield, followed by staining with PE-conjugated anti-Foxp3 antibody for 30 minutes using Foxp3 Staining Buffer Set (eBioscience) according to the manufacture’s protocol for cell fixation and permeabilization (3). For Th17 staining, cells were stained with PerCP-conjugated anti-CD4 and FITC-conjugated anti-CD8a antibodies, followed by intracellular staining with PE-conjugated anti-IL17 and APC-conjugated IFN-γ antibodies similar to Treg staining. The stained cells were analyzed on FACSCalibur (BD Bioscience).

*Biomarkers analysis*

Peripheral blood was collected from the retro-orbital plexus, and centrifuged to obtain the blood serum. IL6, IL10, and IL17 levels were measured using commercial available kits (IL6, IL10, IL17, R&D Systems) according to the manufacturer’ instructions. To measure total TGF-β1, acid-treated samples were analyzed using a kit (Promega) according to the manufacturer’s instruction. Serum creatinine levels were assayed using a commercial kit (R&D Systems). The results were averaged in each group. The intra-group differences were calculated between the mean values. ELISA kit for C reactive protein (CRP) was purchased from Innovative Research Inc. (Novi, MI) and measured according to the manufactures’ instructions.

**REFERENCE**

1.Yamaza, T. *et al*. 2008 Pharmacologic stem cell based intervention as a new approach to osteoporosis treatment in rodents. PLoS ONE **9**, 3: e2615

2.Yamaza, T. *et al*. 2009 Mesenchymal stem cell-mediated ectopic hematopoiesis alleviates aging-related phenotype in immunocompromised mice. Blood **113**: 2595-2604.

3. Liu, Y. et al., 2008 A critical function for TGF-beta signaling in the development of natural CD4+CD25+Foxp3+ regulatory T cells. Nat Immunol **9:**632-640.
